# Supplementary material for: Transcriptomic landscape of posterior regeneration in the annelid Platynereis dumerilii
Source: BMC Genomics. 2023 Oct 2;24:583. doi: 10.1186/s12864-023-09602-z (PMC10546743; doi:10.1186/s12864-023-09602-z)
Supplement: Supplementary file 1 — Additional file 1. [file 12864_2023_9602_MOESM1_ESM.pdf]

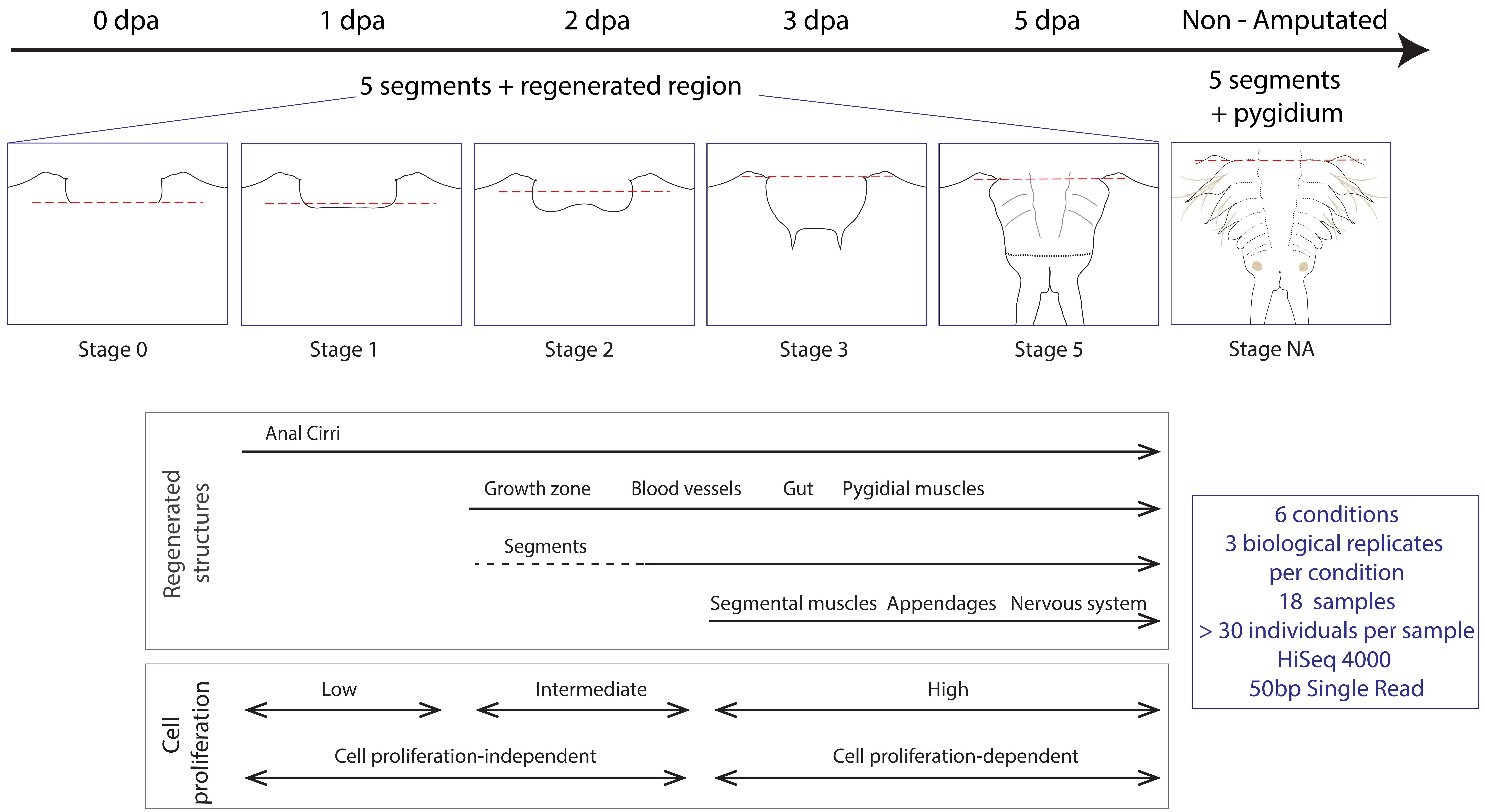

Additional file 1: Experimental design for the transcriptomic analysis of regeneration

Schematic representation of the five stages of regeneration (stage 0 to stage 5) and the non-amputated control, as well as their corresponding days post amputation (dpa, from 0 to 5) are shown. The amputation plan is represented by a red dotted-line on each schematic. From stage 0 to stage 5, the regenerated region (if any) as well as the 5 anterior segments were collected (around 30 individuals per stage) for the RNA-seq experiment. Experimental information is provided in blue on the right. In addition, main morphological and cellular steps during the course of regeneration are shown at the bottom.
