# Supplementary material for: Transcriptomic landscape of posterior regeneration in the annelid Platynereis dumerilii
Source: BMC Genomics. 2023 Oct 2;24:583. doi: 10.1186/s12864-023-09602-z (PMC10546743; doi:10.1186/s12864-023-09602-z)
Supplement: Supplementary file 3 — Additional file 3. [file 12864_2023_9602_MOESM3_ESM.pdf]

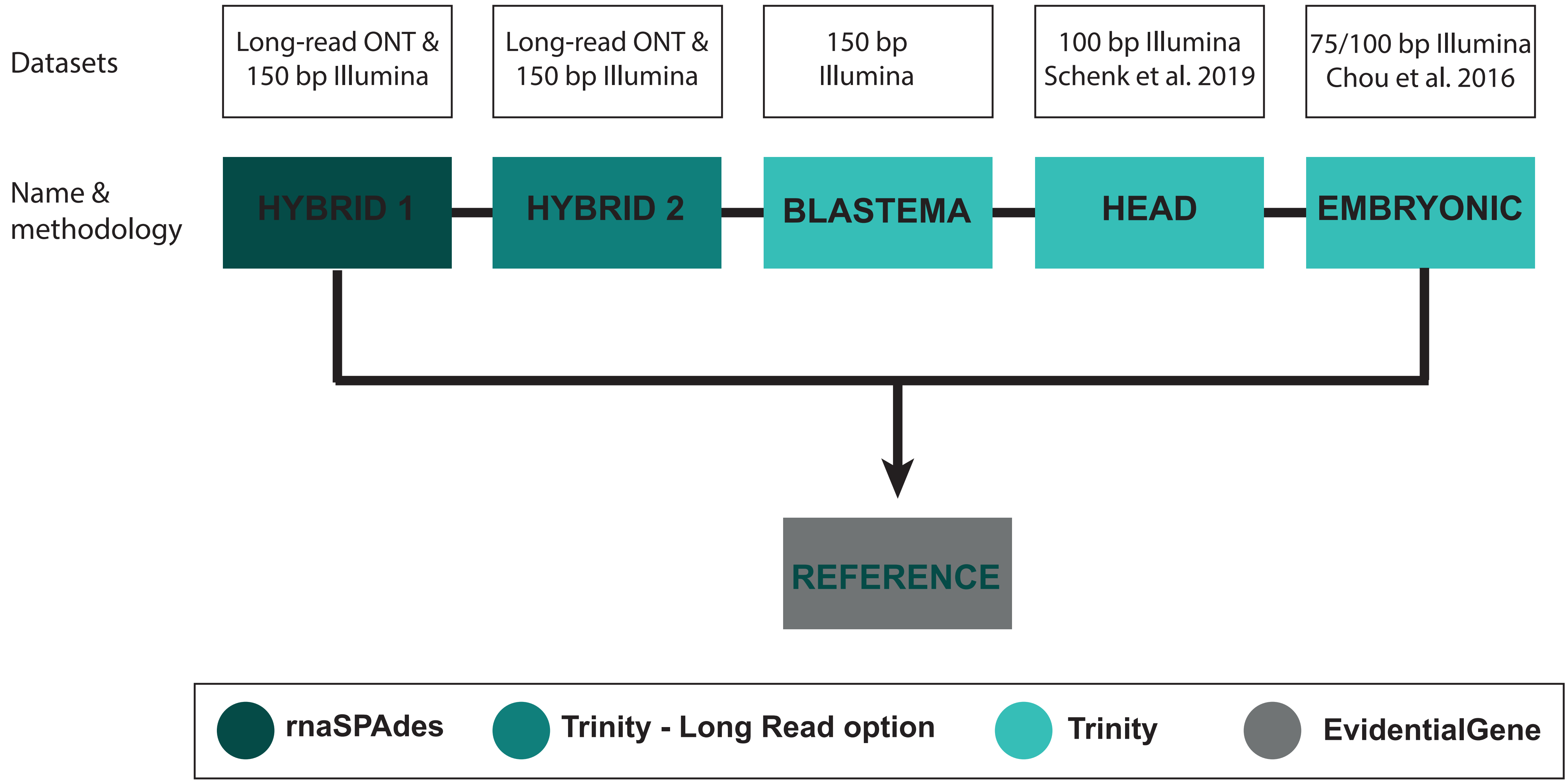

Additional file 3: Reference transcriptome assembly strategy

Schematic representation of the transcriptome assembly strategy followed in the study. We assembled the Reference by merging three transcriptomes generated in this study (Hybrid 1, Hybrid 2 and Blastema) that were assembled using different combinations of data and methods (see Methods and Results and Discussion), and two transcriptomes extracted from the literature: Head (45) and Embryonic (44). Merging transcriptomes was performed using EvidentialGene (92). The type of data used per transcriptome assembly is mentioned on the top line of the schematic, while the different assemblers are color-coded and their names mentioned at the bottom of the schematic.
