# Supplementary material for: Transcriptomic landscape of posterior regeneration in the annelid Platynereis dumerilii
Source: BMC Genomics. 2023 Oct 2;24:583. doi: 10.1186/s12864-023-09602-z (PMC10546743; doi:10.1186/s12864-023-09602-z)
Supplement: Supplementary file 8 — Additional file 8. [file 12864_2023_9602_MOESM8_ESM.pdf]

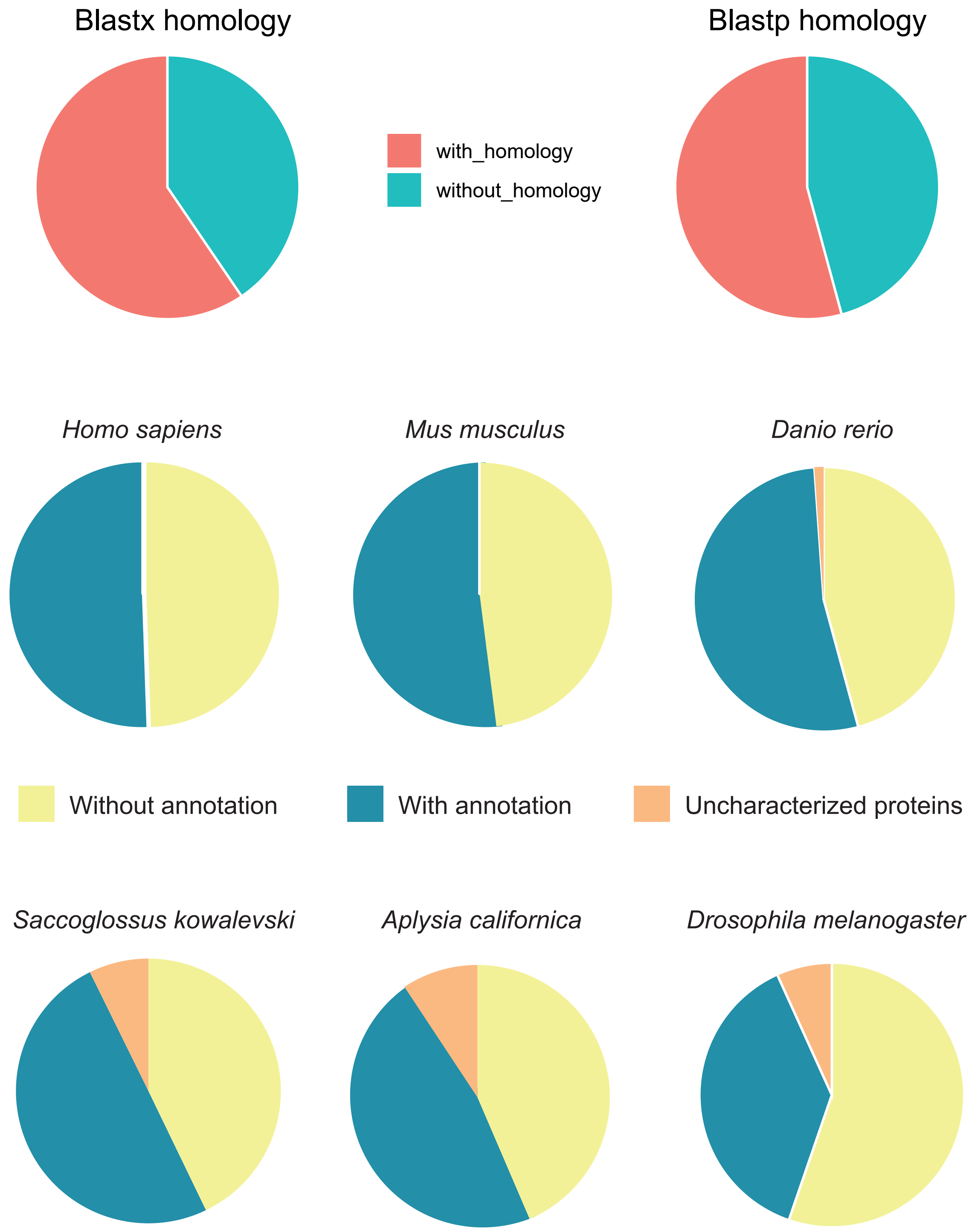

Additional file 8: BLAST results for the Reference transcriptome
Top panel: Graphic representation of BLASTX and BLASTP results from Trinotate (see Additional file 7). Areas in red and blue represent the percentage of *P. dumerilii* transcripts with (respectively without) a significant BLAST hit. Middle and bottom panels: Graphic representation of BLASTX results between our Reference transcriptome and six bilaterian species (*H. sapiens, M. musculus, D. rerio, S. kowalevski, A. californica, D. melanogaster*). Areas in green, orange and yellow represent the percentage of *P. dumerilii* transcripts with a significant BLAST hit on an annotated protein, with a significant BLAST hit on an uncharacterized protein, and without a significant blast hit (e-value cutoff = 10-3) respectively.
