# Supplementary material for: Transcriptomic landscape of posterior regeneration in the annelid Platynereis dumerilii
Source: BMC Genomics. 2023 Oct 2;24:583. doi: 10.1186/s12864-023-09602-z (PMC10546743; doi:10.1186/s12864-023-09602-z)
Supplement: Supplementary file 10 — Additional file 10. [file 12864_2023_9602_MOESM10_ESM.pdf]

A With outliers

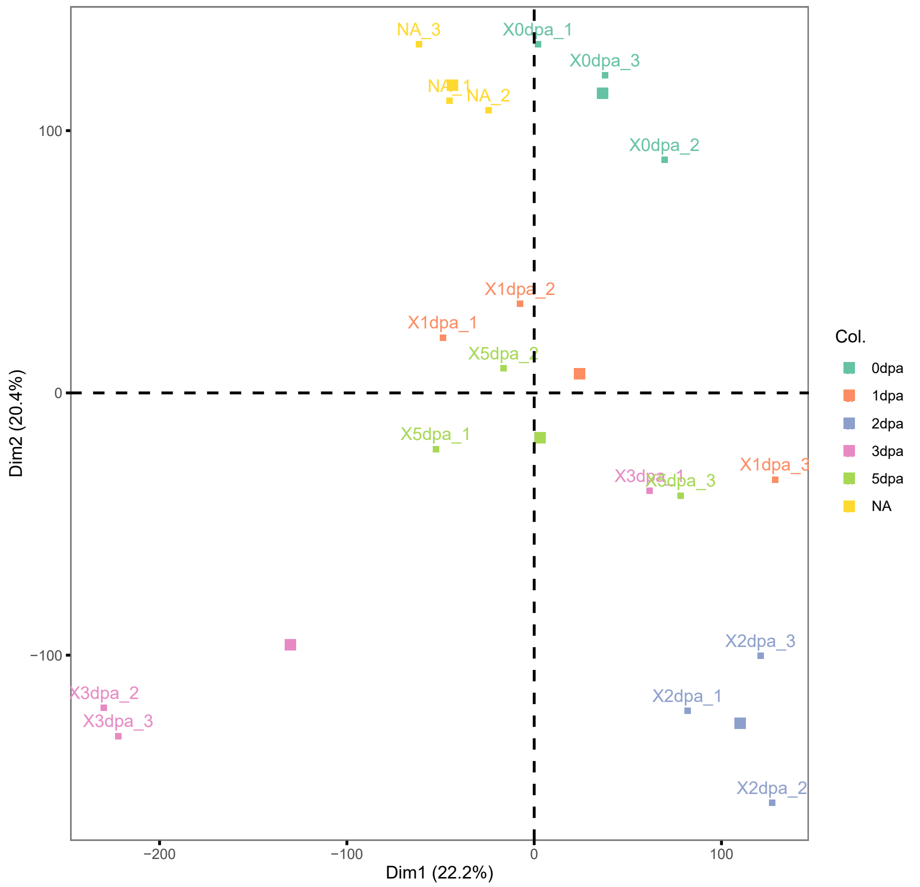

B Without outliers

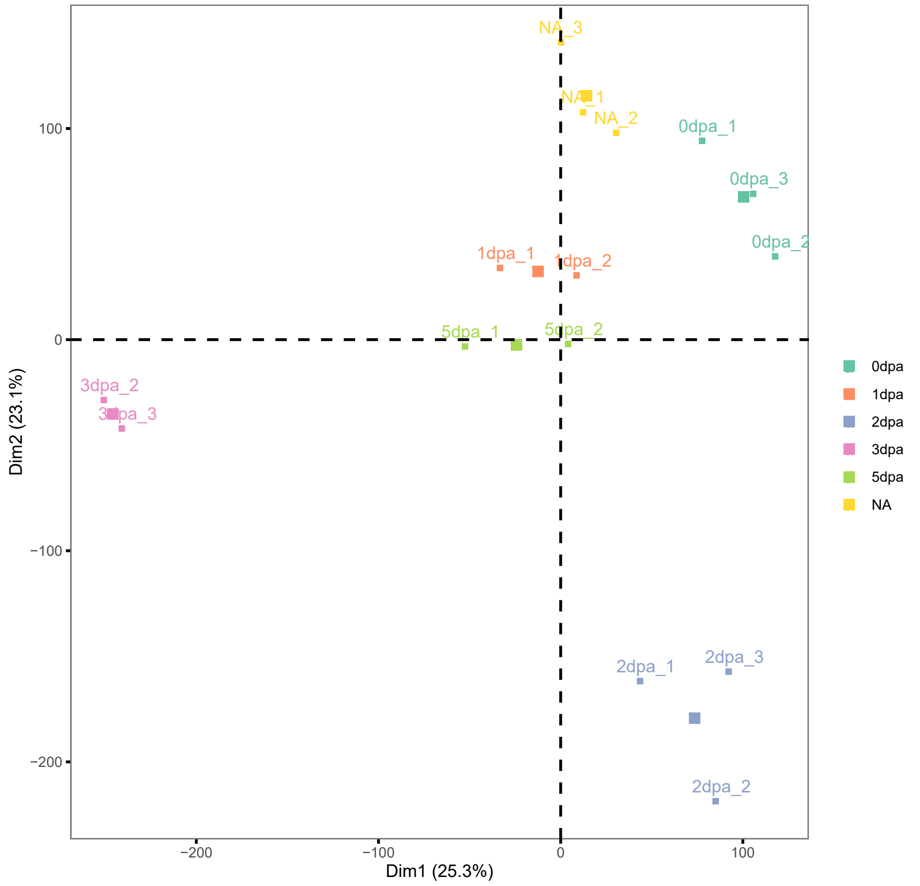

Additional file 10: PCA plot representing the relatedness of RNA-seq samples obtained for six regeneration stages

A) PCA with all samples, B) Outlying samples have been removed (see Main Text for details). Two to three replicates per stages are present. Each dot represents an RNA-seq sample. Colors represent biological conditions (*i.e.* regeneration stages and non-amputated worms, indicated on the right), biological replicates are also indicated (by a number on the name of the samples).
