# Supplementary material for: Transcriptomic landscape of posterior regeneration in the annelid Platynereis dumerilii
Source: BMC Genomics. 2023 Oct 2;24:583. doi: 10.1186/s12864-023-09602-z (PMC10546743; doi:10.1186/s12864-023-09602-z)
Supplement: Supplementary file 19 — Additional file 19. [file 12864_2023_9602_MOESM19_ESM.pdf]

Additional file 19: GO term analysis of gene expression clusters

Treeplot representation of clustered enriched GO term (Biological process) analysis for 8 clusters (1, 2, 4, 7, 8, 9, 10 and 11). Circles area are proportional to the number of genes in each annotation, the colors represent the p-value of the over-representation.

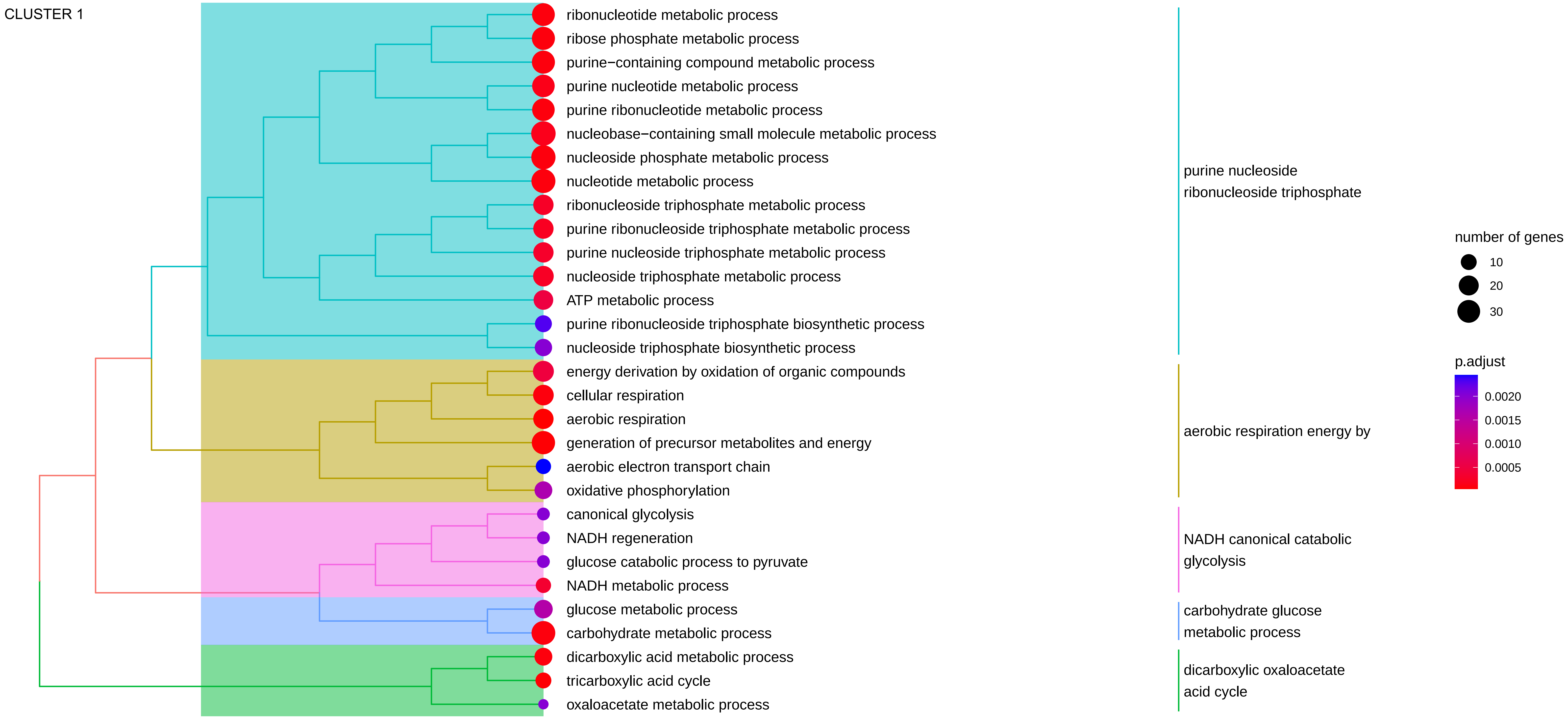
CLUSTER 1
ribonucleotide metabolic process
ribose phosphate metabolic process
purine−containing compound metabolic process
purine nucleotide metabolic process
purine ribonucleotide metabolic process
nucleobase−containing small molecule metabolic process
nucleoside phosphate metabolic process
nucleotide metabolic process
ribonucleoside triphosphate metabolic process
purine ribonucleoside triphosphate metabolic process
purine nucleoside triphosphate metabolic process
nucleoside triphosphate metabolic process
ATP metabolic process
purine ribonucleoside triphosphate biosynthetic process
nucleoside triphosphate biosynthetic process
energy derivation by oxidation of organic compounds
cellular respiration
aerobic respiration
generation of precursor metabolites and energy
aerobic electron transport chain
oxidative phosphorylation
canonical glycolysis
NADH regeneration
glucose catabolic process to pyruvate
NADH metabolic process
glucose metabolic process
carbohydrate metabolic process
dicarboxylic acid metabolic process
tricarboxylic acid cycle
oxaloacetate metabolic process
purine nucleoside ribonucleoside triphosphate
aerobic respiration energy by
NADH canonical catabolic glycolysis
carbohydrate glucose metabolic process
dicarboxylic oxaloacetate acid cycle
number of genes
10
20
30
p.adjust
0.0020
0.0015
0.0010
0.0005

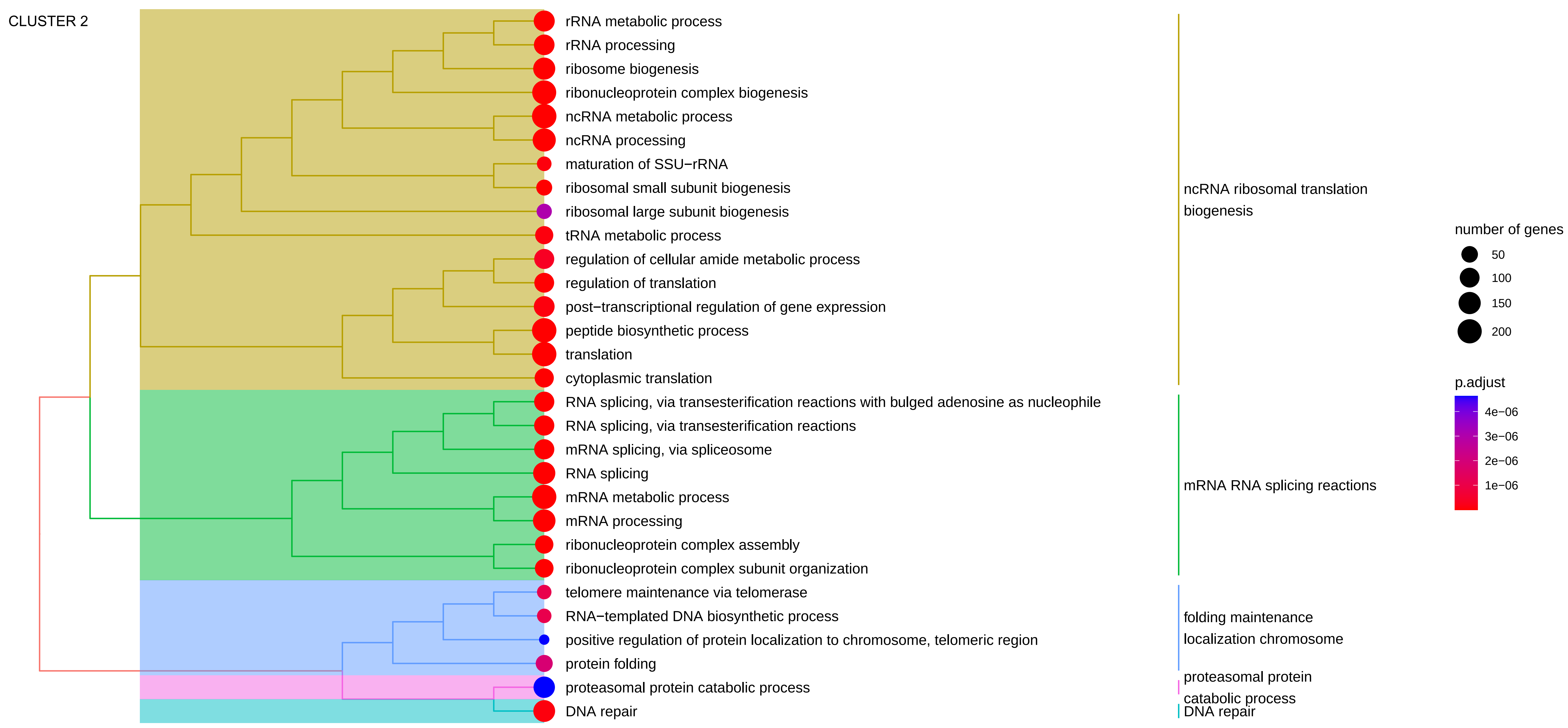
CLUSTER 2
rRNA metabolic process
rRNA processing
ribosome biogenesis
ribonucleoprotein complex biogenesis
ncRNA metabolic process
ncRNA processing
maturation of SSU−rRNA
ribosomal small subunit biogenesis
ribosomal large subunit biogenesis
tRNA metabolic process
regulation of cellular amide metabolic process
regulation of translation
post−transcriptional regulation of gene expression
peptide biosynthetic process
translation
cytoplasmic translation
RNA splicing, via transesterification reactions with bulged adenosine as nucleophile
RNA splicing, via transesterification reactions
mRNA splicing, via spliceosome
RNA splicing
mRNA metabolic process
mRNA processing
ribonucleoprotein complex assembly
ribonucleoprotein complex subunit organization
telomere maintenance via telomerase
RNA−templated DNA biosynthetic process
positive regulation of protein localization to chromosome, telomeric region
protein folding
proteasomal protein catabolic process
DNA repair
ncRNA ribosomal translation biogenesis
mRNA RNA splicing reactions
folding maintenance localization chromosome
proteasomal protein catabolic process
DNA repair
number of genes
50
100
150
200
p.adjust
4e−06
3e−06
2e−06
1e−06

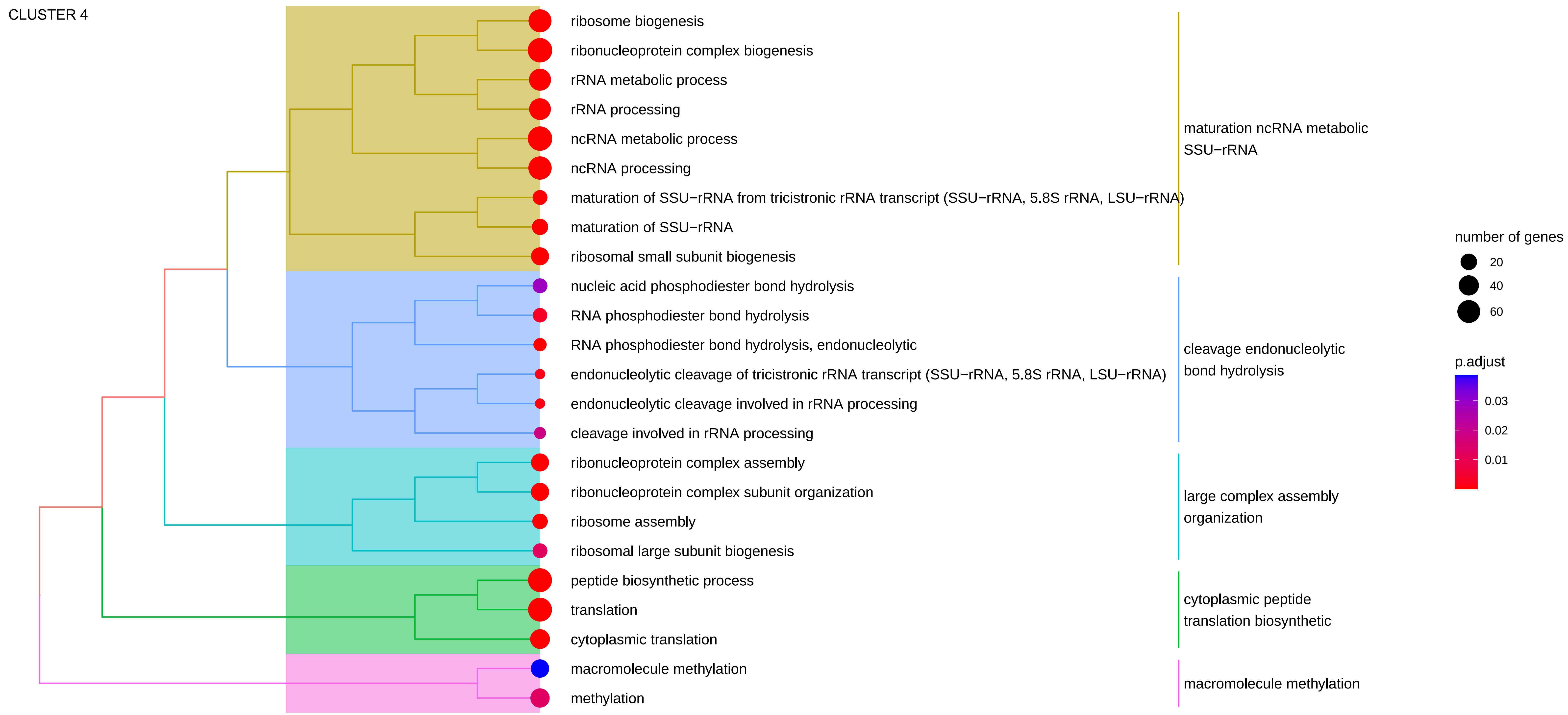
CLUSTER 4
ribosome biogenesis
ribonucleoprotein complex biogenesis
rRNA metabolic process
rRNA processing
ncRNA metabolic process
ncRNA processing
maturation of SSU−rRNA from tricistronic rRNA transcript (SSU−rRNA, 5.8S rRNA, LSU−rRNA)
maturation of SSU−rRNA
ribosomal small subunit biogenesis
nucleic acid phosphodiester bond hydrolysis
RNA phosphodiester bond hydrolysis
RNA phosphodiester bond hydrolysis, endonucleolytic
endonucleolytic cleavage of tricistronic rRNA transcript (SSU−rRNA, 5.8S rRNA, LSU−rRNA)
endonucleolytic cleavage involved in rRNA processing
cleavage involved in rRNA processing
ribonucleoprotein complex assembly
ribonucleoprotein complex subunit organization
ribosome assembly
ribosomal large subunit biogenesis
peptide biosynthetic process
translation
cytoplasmic translation
macromolecule methylation
methylation
maturation ncRNA metabolic SSU−rRNA
cleavage endonucleolytic bond hydrolysis
large complex assembly organization
cytoplasmic peptide translation biosynthetic
macromolecule methylation
number of genes
20
40
60
p.adjust
0.03
0.02
0.01

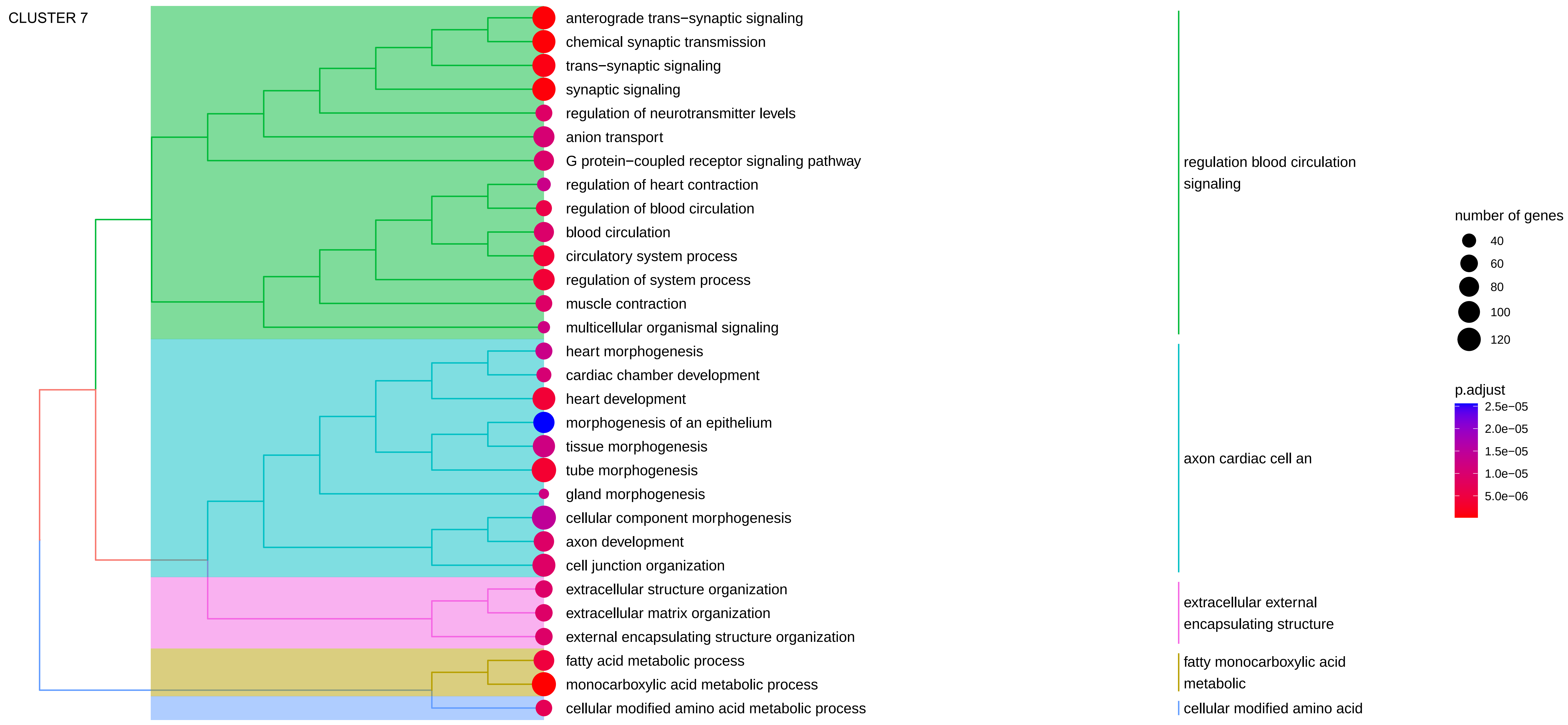
CLUSTER 7
anterograde trans−synaptic signaling
chemical synaptic transmission
trans−synaptic signaling
synaptic signaling
regulation of neurotransmitter levels
anion transport
G protein−coupled receptor signaling pathway
regulation of heart contraction
regulation of blood circulation
blood circulation
circulatory system process
regulation of system process
muscle contraction
multicellular organismal signaling
heart morphogenesis
cardiac chamber development
heart development
morphogenesis of an epithelium
tissue morphogenesis
tube morphogenesis
gland morphogenesis
cellular component morphogenesis
axon development
cell junction organization
extracellular structure organization
extracellular matrix organization
external encapsulating structure organization
fatty acid metabolic process
monocarboxylic acid metabolic process
cellular modified amino acid metabolic process
regulation blood circulation signaling
axon cardiac cell an
extracellular external encapsulating structure
fatty monocarboxylic acid metabolic
cellular modified amino acid
number of genes
40
60
80
100
120
p.adjust
2.5e−05
2.0e−05
1.5e−05
1.0e−05
5.0e−06

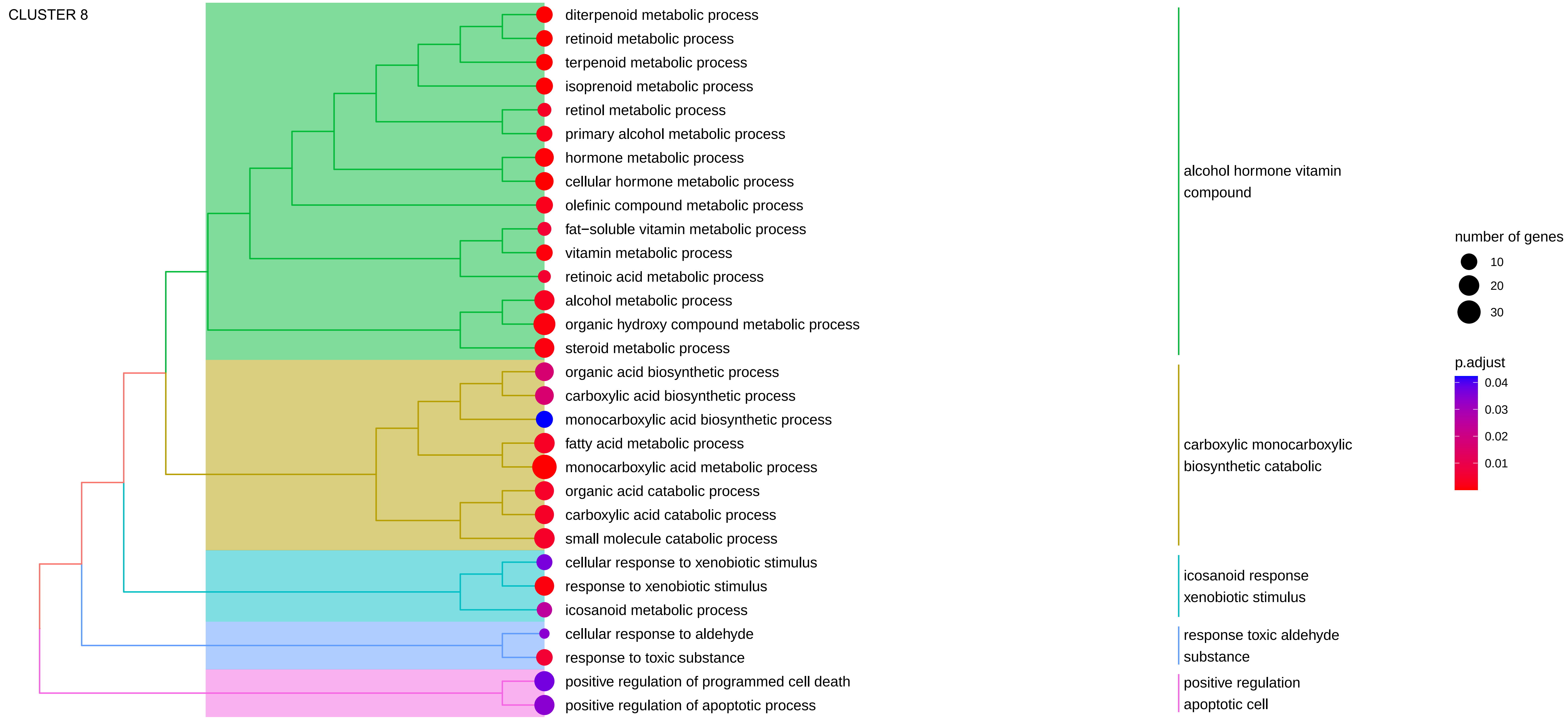
CLUSTER 8
diterpenoid metabolic process
retinoid metabolic process
terpenoid metabolic process
isoprenoid metabolic process
retinol metabolic process
primary alcohol metabolic process
hormone metabolic process
cellular hormone metabolic process
olefinic compound metabolic process
fat−soluble vitamin metabolic process
vitamin metabolic process
retinoic acid metabolic process
alcohol metabolic process
organic hydroxy compound metabolic process
steroid metabolic process
organic acid biosynthetic process
carboxylic acid biosynthetic process
monocarboxylic acid biosynthetic process
fatty acid metabolic process
monocarboxylic acid metabolic process
organic acid catabolic process
carboxylic acid catabolic process
small molecule catabolic process
cellular response to xenobiotic stimulus
response to xenobiotic stimulus
icosanoid metabolic process
cellular response to aldehyde
response to toxic substance
positive regulation of programmed cell death
positive regulation of apoptotic process
alcohol hormone vitamin compound
carboxylic monocarboxylic biosynthetic catabolic
icosanoid response xenobiotic stimulus
response toxic aldehyde substance
positive regulation apoptotic cell
number of genes
10
20
30
p.adjust
0.04
0.03
0.02
0.01

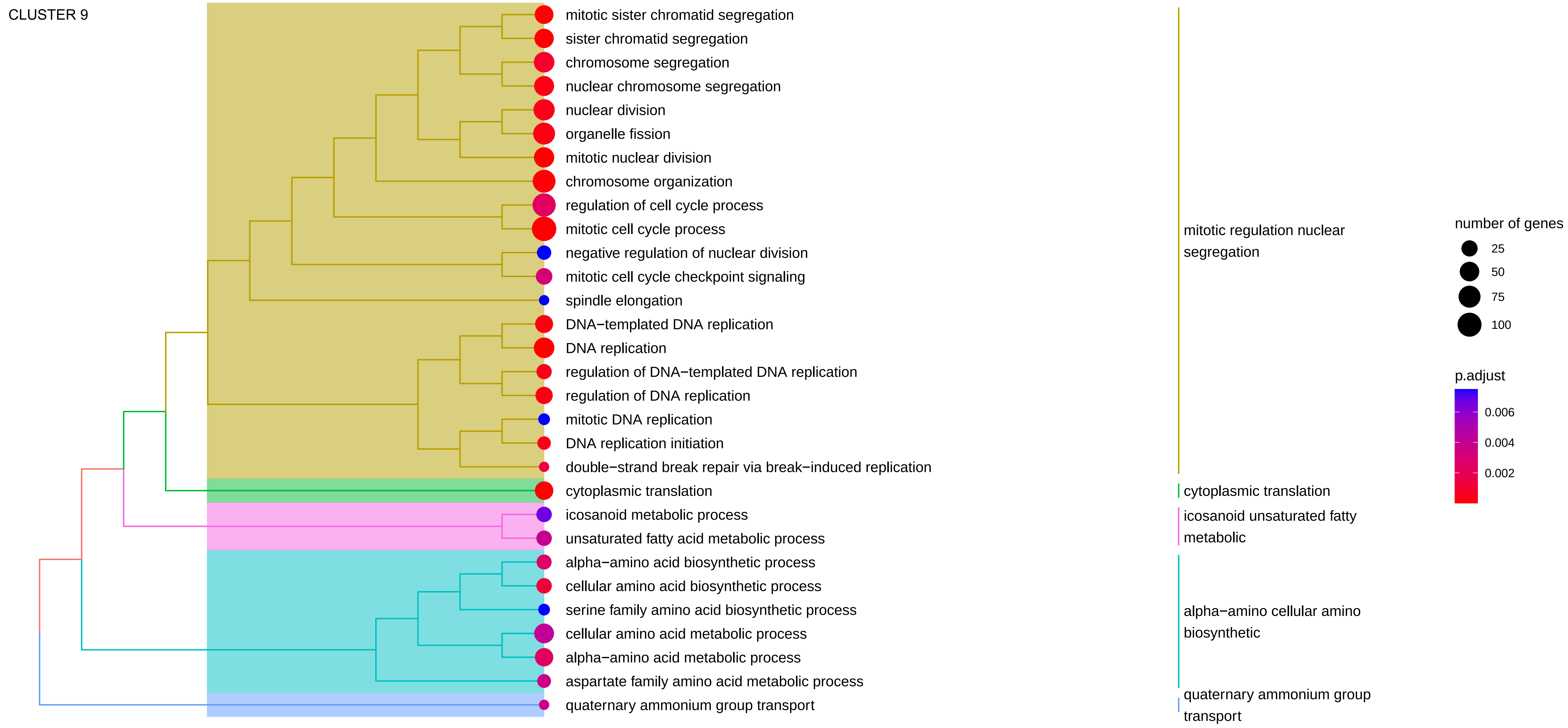
CLUSTER 9
mitotic sister chromatid segregation
sister chromatid segregation
chromosome segregation
nuclear chromosome segregation
nuclear division
organelle fission
mitotic nuclear division
chromosome organization
regulation of cell cycle process
mitotic cell cycle process
negative regulation of nuclear division
mitotic cell cycle checkpoint signaling
spindle elongation
DNA−templated DNA replication
DNA replication
regulation of DNA−templated DNA replication
regulation of DNA replication
mitotic DNA replication
DNA replication initiation
double−strand break repair via break−induced replication
cytoplasmic translation
icosanoid metabolic process
unsaturated fatty acid metabolic process
alpha−amino acid biosynthetic process
cellular amino acid biosynthetic process
serine family amino acid biosynthetic process
cellular amino acid metabolic process
alpha−amino acid metabolic process
aspartate family amino acid metabolic process
quaternary ammonium group transport
mitotic regulation nuclear segregation
cytoplasmic translation
icosanoid unsaturated fatty metabolic
alpha−amino cellular amino biosynthetic
quaternary ammonium group transport
number of genes
25
50
75
100
p.adjust
0.006
0.004
0.002

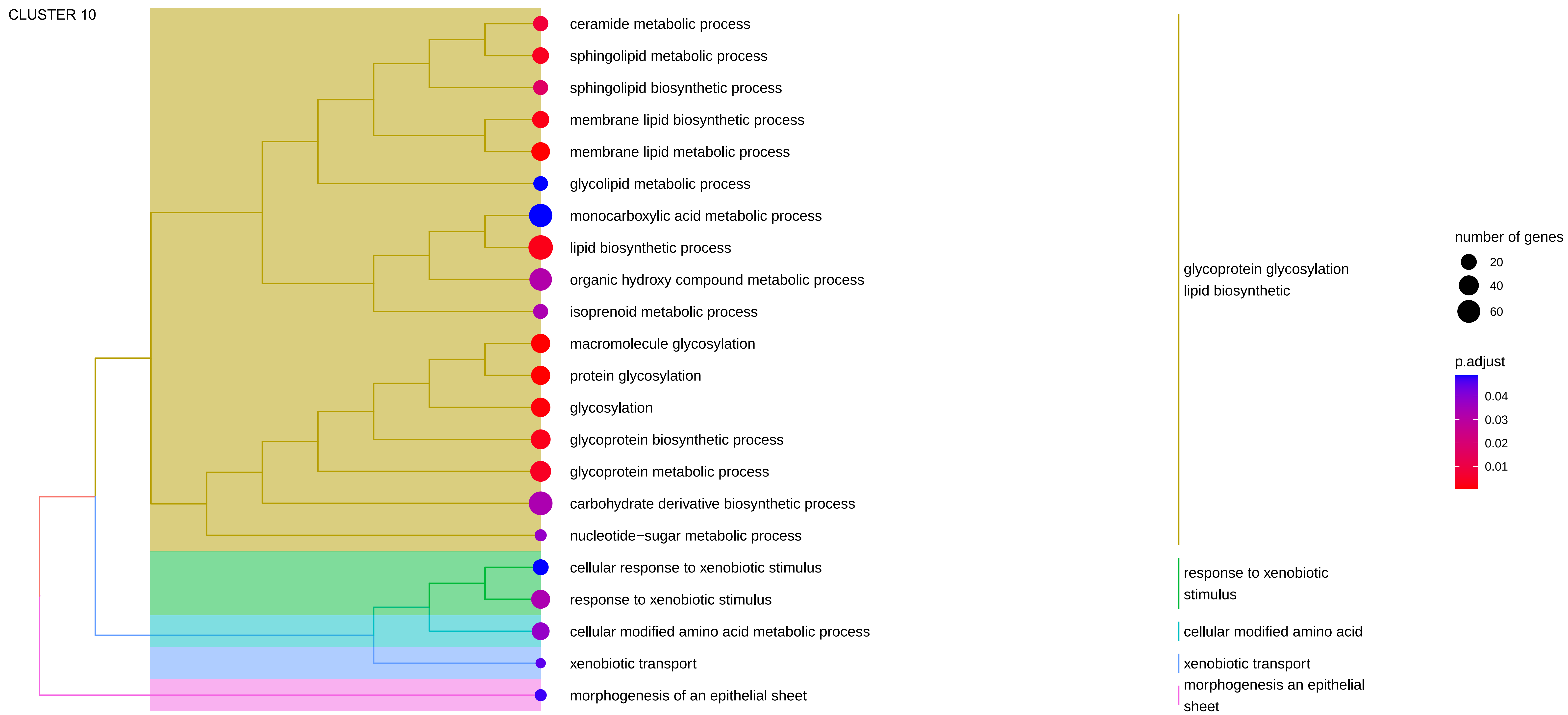
CLUSTER 10
ceramide metabolic process
sphingolipid metabolic process
sphingolipid biosynthetic process
membrane lipid biosynthetic process
membrane lipid metabolic process
glycolipid metabolic process
monocarboxylic acid metabolic process
lipid biosynthetic process
organic hydroxy compound metabolic process
isoprenoid metabolic process
macromolecule glycosylation
protein glycosylation
glycosylation
glycoprotein biosynthetic process
glycoprotein metabolic process
carbohydrate derivative biosynthetic process
nucleotide−sugar metabolic process
cellular response to xenobiotic stimulus
response to xenobiotic stimulus
cellular modified amino acid metabolic process
xenobiotic transport
morphogenesis of an epithelial sheet
glycoprotein glycosylation
lipid biosynthetic
response to xenobiotic
stimulus
cellular modified amino acid
xenobiotic transport
morphogenesis an epithelial
sheet
number of genes
20
40
60
p.adjust
0.04
0.03
0.02
0.01

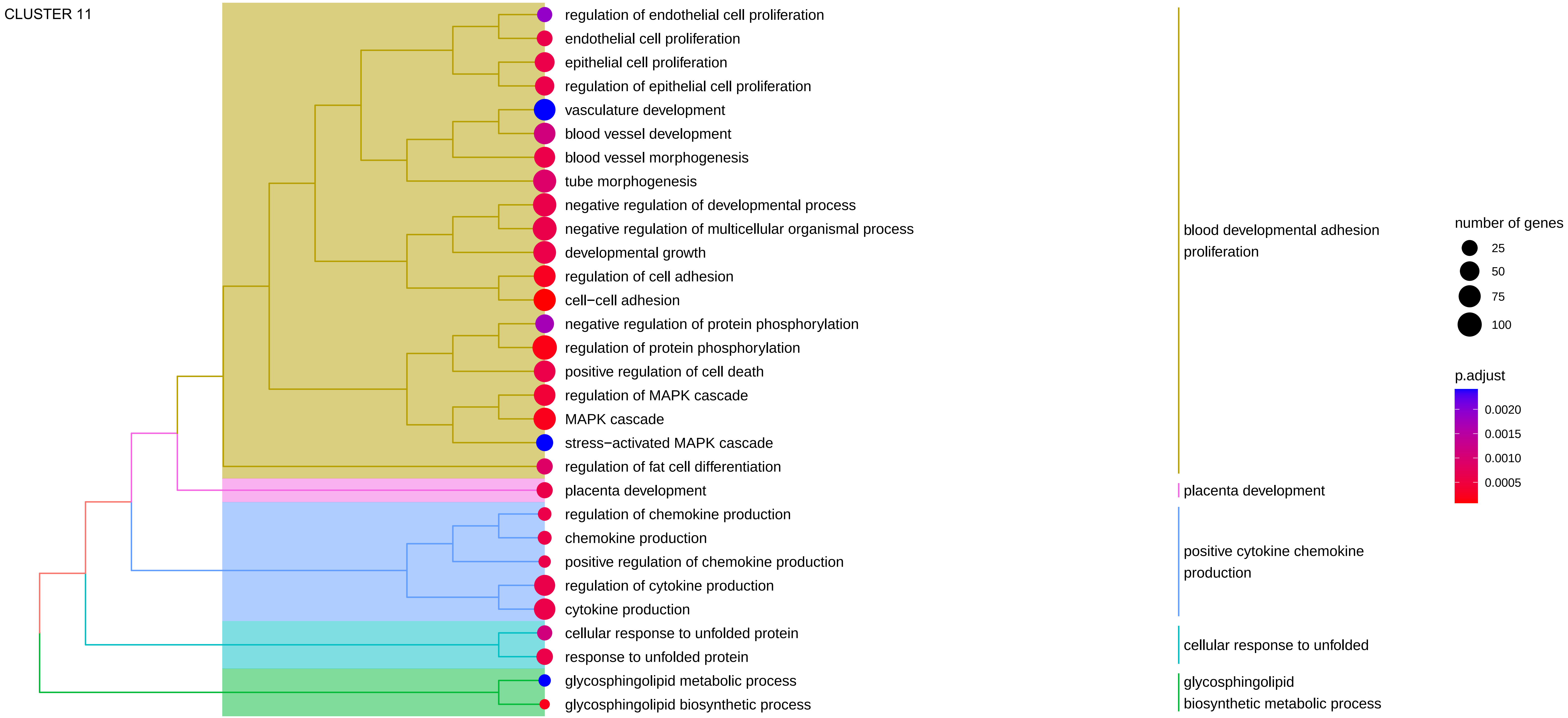
CLUSTER 11
regulation of endothelial cell proliferation
endothelial cell proliferation
epithelial cell proliferation
regulation of epithelial cell proliferation
vasculature development
blood vessel development
blood vessel morphogenesis
tube morphogenesis
negative regulation of developmental process
negative regulation of multicellular organismal process
developmental growth
regulation of cell adhesion
cell−cell adhesion
negative regulation of protein phosphorylation
regulation of protein phosphorylation
positive regulation of cell death
regulation of MAPK cascade
MAPK cascade
stress−activated MAPK cascade
regulation of fat cell differentiation
placenta development
regulation of chemokine production
chemokine production
positive regulation of chemokine production
regulation of cytokine production
cytokine production
cellular response to unfolded protein
response to unfolded protein
glycosphingolipid metabolic process
glycosphingolipid biosynthetic process
blood developmental adhesion proliferation
placenta development
positive cytokine chemokine production
cellular response to unfolded
glycosphingolipid biosynthetic metabolic process
number of genes
25
50
75
100
p.adjust
0.0020
0.0015
0.0010
0.0005
